# Leukemia mortality in children from Latin America: trends and predictions to 2030

**DOI:** 10.1186/s12887-020-02408-y

**Published:** 2020-11-07

**Authors:** J. Smith Torres-Roman, Bryan Valcarcel, Pedro Guerra-Canchari, Camila Alves Dos Santos, Isabelle Ribeiro Barbosa, Carlo La Vecchia, Katherine A. McGlynn, Dyego Leandro Bezerra de Souza

**Affiliations:** 1 Universidad Científica del Sur, Lima, Peru; 2 Universidad Católica Los Ángeles de Chimbote, Instituto de Investigación, Chimbote, Peru; 3 Latin American Network for Cancer Research (LAN–CANCER), Lima, Peru; 4 Milken Institute School of Public Health, The George Washington University, Washington, DC USA; 5 Sociedad Científica San Fernando, Universidad Nacional Mayor de San Marcos, Lima, Peru; 6 Graduate Program in Collective Health, Federal University of Rio Grande do Norte, Natal, Rio Grande do Norte State Brazil; 7 Department of Clinical Sciences and Community Health, Università degli Studi di Milano, 20133 Milan, Italy; 8 Division of Cancer Epidemiology and Genetics, National Cancer Institute, Bethesda, MD USA; 9 Research group on Methodology, Methods, Models and Outcomes of Health and Social Sciences (M3O), Faculty of Health Sciences and Welfare, Centre for Health and Social Care Research (CESS), University of Vic-Central University of Catalonia (UVic-UCC), Barcelona, Spain

**Keywords:** Child, Leukemia, Mortality rate, Trends, epidemiology, Latin America

## Abstract

**Background:**

Reports suggest that Latin American and Caribbean (LAC) countries have not reduced leukemia mortality compared to high-income countries. However, updated trends remain largely unknown in the region. Given that leukemia is the leading cause of cancer-related death in LAC children, we evaluated mortality trends in children (0-14y) from 15 LAC countries for the period 2000–2017 and predicted mortality to 2030.

**Methods:**

We retrieved cancer mortality data using the World Health Organization Mortality Database. Mortality rates (standardized to the world standard SEGI population) were analyzed for 15 LAC countries. We evaluated the average mortality rates for the last 5 years (2013–2017). Joinpoint regression analysis was used to evaluate leukemia mortality trends and provide an estimated annual percent change (EAPC). Nordpred was utilized for the calculation of predictions until 2030.

**Results:**

Between 2013 and 2017, the highest mortality rates were reported in Venezuela, Ecuador, Nicaragua, Mexico, and Peru. Upward mortality trends were reported in Nicaragua (EAPC by 2.9% in boys, and EAPC by 2.0% in girls), and Peru (EAPC by 1.4% in both sexes). Puerto Rico experienced large declines in mortality among both boys (EAPC by − 9.7%), and girls (EAPC by − 6.0%). Leukemia mortality will increase in Argentina, Ecuador, Guatemala, Panama, Peru, and Uruguay by 2030.

**Conclusion:**

Leukemia mortality is predicted to increase in some LAC countries by 2030. Interventions to prevent this outcome should be tailor to reduce the socioeconomic inequalities and ensure universal healthcare coverage.

**Supplementary Information:**

The online version contains supplementary material available at 10.1186/s12887-020-02408-y.

## Background

Leukemia is a heterogeneous group of hematologic malignancies [1]. In 2018, GLOBOCAN reported nearly 65,000 new cases (incidence rate of 3.4 per 100,000) and approximately 30,000 deaths (mortality rate of 1.5 per 100,000) in children under 15 of age worldwide [2, 3]. Both incidence and mortality are higher in boys compared to girls [1, 3]. In fact, GLOBOCAN reported that the incidence rate among boys was 3.8 per 100,000 and the mortality rate was 1.7 per 100,000, whereas the incidence rate among girls was 2.9 per 100,000 and the mortality rate was 1.3 per 100,000 [3]. Regarding childhood leukemia, acute lymphoblastic leukemia (80%) is the most common subtype, followed by acute myeloid leukemia (15%), and other types of leukemia (5%) [1, 4].

Over the last several years, many countries have experienced declining childhood leukemia mortality rates, principally due to the improvement of treatment regimens and supportive care [5–8]. For example, a study in the European children reported a decrease among boys mortality rates from 1.35 in 1997 to 0.85 in 2007 (37% reduction), whereas among girls declined from 1.07 in 1997 to 0.70 in 2007 (35% reduction) [5].

Notwithstanding these improvements, leukemia remains the leading cause of cancer mortality in Latin American and the Caribbean (LAC) children, considered as a major public health challenge in this region [9, 10]. For example, between 1980 and 2014, Mexico reported a rise of mortality rates of leukemia (19% in boys and 21% in girls) [10]. The barriers to healthcare access, social disparities, and the lack of economic resources in LAC hamper the survival improvements seen in high-income countries [11, 12]. Other factors related to increased mortality include delayed diagnosis, treatment abandonment, lack of a proper supportive care, and a shortage of pediatric oncologists as well as nurses dedicated to pediatric oncology care [13, 14].

To our knowledge, few studies have attempted to provide a comprehensive population-based analysis of leukemia mortality trends in the LAC region [10]. Given the high mortality burden among LAC countries, it is important to perform updated analysis to identify and evaluate the current status of leukemia mortality for the future development of evidence-based health policies. Along this line, the use of cancer predictions provides further insight toward optimal decision making in public health and the correct planning and allocation of resources for health improvement. Therefore, we sought to identify mortality trends for leukemia in children from LAC countries between 2000 and 2017 and to predict mortality until 2030.

## Methods

### Design and study setting

A study of mortality trends was conducted based on data from the World Health Organization Mortality Database [15]. The countries that had data available for analysis between 2000 and 2017 included: Argentina, Brazil, Chile, Costa Rica, Cuba, Ecuador, Guatemala, Mexico, Nicaragua, Panama, Paraguay, Peru, Puerto Rico, Uruguay, and Venezuela (2000–2014). We included deaths due to leukemia (C91-C95) according to the International Statistical Classification of Diseases and Related Health Problems – 10th revision [16]. Population demographics (sex and age) were obtained for each country from the Pan American Health Organization [17], as previous studies in Latin America have done [18, 19].

### Data analysis

**Fig. 1:**
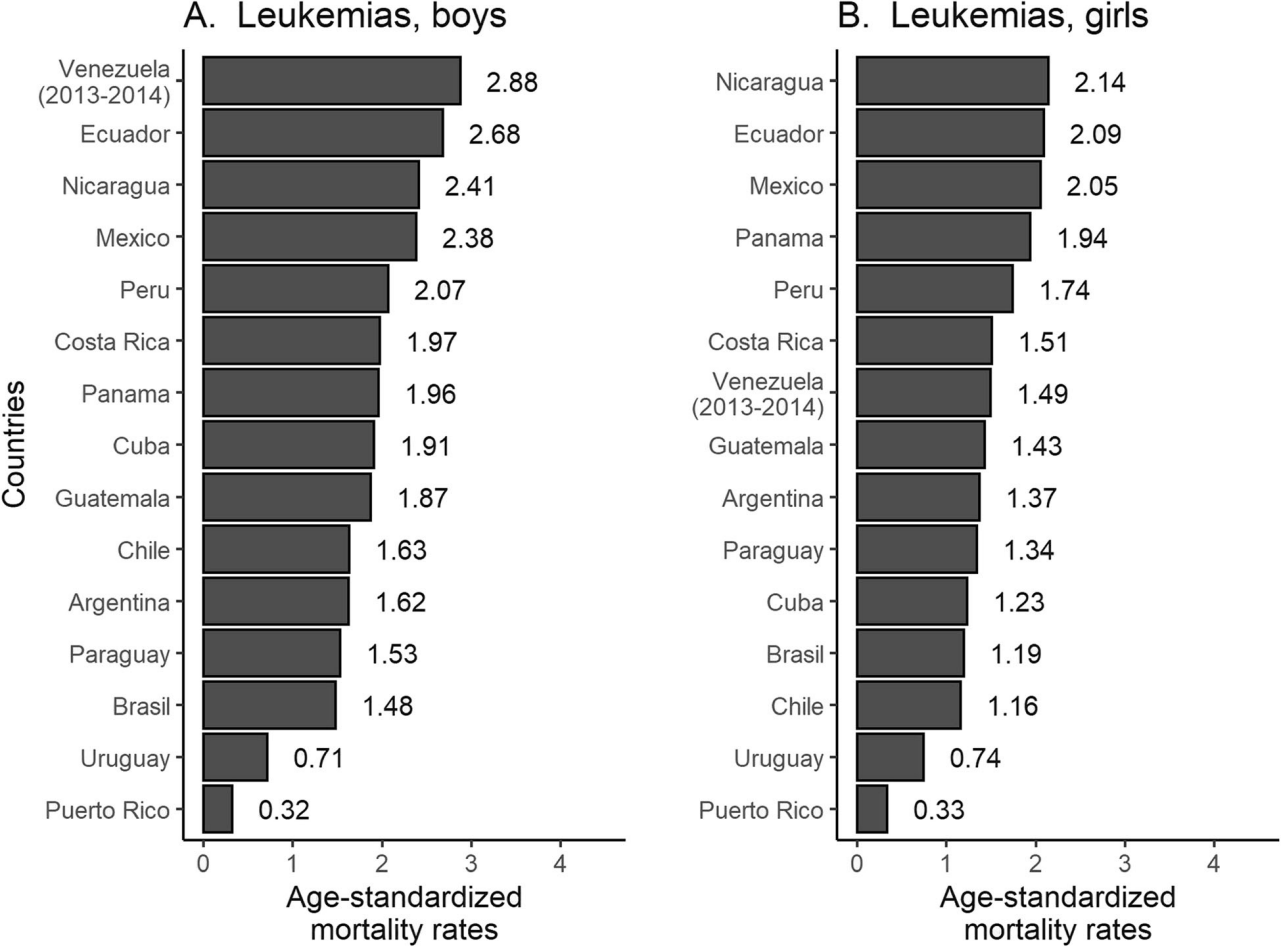
Age-standardized leukemia mortality rates (world standard population) per 100,000 among boys and girls 0–14 years of age from 15 Latin American countries between 2013 and 2017

Age-standardized mortality rates (ASMRs) were estimated using the direct method and the world standard SEGI population per 100,000 persons-years [20]. Leukemia mortality trends were analyzed for children aged 0 to 14 years, stratified in three age groups (0–4, 5–9, and 10–14) between 2000 and 2017. We computed the average mortality rates for the last 5 years with the purpose of showing updated data for leukemia in LAC (Fig. 1). We also provided an estimate of overall leukemia mortality rates in the LAC for the year 2030.

Mortality trends analysis was carried out by Joinpoint regression, utilizing the *Joinpoint Regression Program*, version 4.6.0.0 (National Cancer Institute, Bethesda, Maryland, USA) [21]. The objective of the analysis was to identify the years where a significant change in the linear slope of the trend (on a log-scale) was detected over the study period. The method identified a maximum of three joinpoints. The final selected model provided the estimated annual percentage change (EAPC) based on the trend of each segment, using a 95% confidence interval. The significance levels utilized were based on the Monte Carlo permutation model and on the calculation of the annual percentage change of ratio, utilizing the logarithm of the ratio [22, 23].

Predictions were made for each period utilizing the age-period-cohort model from the Nordpred program (Cancer Registry of Norway, Oslo, Norway), using the R software program. Data were compiled in blocks of five years and the limit age group considered for analysis was the first with more than 10 cases for the combined period [24, 25]. This model allows the comparison of birth cohorts, calculated by the decrease in the age range by calendar period, which result in intervals for the years of birth of each 5-year cohort. The calculation can be represented by the following formula [25]:

$$ {\mathrm{R}}_{\mathrm{a}\mathrm{p}}=\exp\ \left({\mathrm{A}}_{\mathrm{a}}+\mathrm{D}\ \mathrm{p}+{\mathrm{P}}_{\mathrm{p}}+{\mathrm{C}}_{\mathrm{c}}\right) $$

In this formula, R_ap_ is the incidence ratio for the age group “a” in the “p” period; D is the common drift parameter (which is the linear average of increase in the observed period); A_a_ is the age-related component for group “a”; P_p_ represents the nonlinear component for the period “p”; and C_c_ corresponds to the nonlinear component of cohort “c”.

The results of the predictions are presented for the total number of deaths observed and expected for each period by country (except for Venezuela due to information available only until 2014). For each period, adjusted mortality rates were calculated based on the world standard SEGI population for global comparisons, expressed per 100,000 persons-years. The predictions of the most recent linear trend for the last ten years was attenuated in the drift parameter of 25% in the second and 50% in third 5-year period prediction periods, and 75% from the fourth period [26]. The objective of this mathematical operation is to reduce the influence of the current trend on predictions. The proposed model is based on empirical comparisons from different methods of predictions [26].

Annual changes were calculated for the number of predicted deaths in 2030 compared to the observed deaths in 2015, where the proportion of the change could occur in terms of changes in risks or demographics (size or structure of the population). These two components can be different from zero and can be either positive or negative in direction. The calculation can be expressed as [26].

$$ \Delta \mathrm{tot}=\Delta \mathrm{risk}+\Delta\ \mathrm{pop}=\left(\mathrm{Nfff}-\mathrm{Noff}\right)+\left(\mathrm{Noff}-\mathrm{Nooo}\right) $$

Where Δtot is the total change, Δrisk is the change in function of risk, Δpop is the change in function of the population, Nooo is the number of observed cases, Nfff is the number of projected cases, and Noff is the number of expected cases when the mortality rates increase during the observed period.

### Ethical considerations

This manuscript is based on administrative databases and does not use any personal identifiable information.

## Results

Figure 1 shows the ASMRs per 100,000 person-years in children from 15 LAC countries between 2013 and 2017. Venezuela, Ecuador, Nicaragua, Mexico, and Peru reported the highest mortality rates in boys (above 2 deaths per 100,000 persons-years), while Nicaragua, Ecuador, and Mexico reported the highest mortality rates in girls (above 2 deaths per 100,000 persons-years). The lowest mortality rates were reported in Uruguay and Puerto Rico for both sexes (below 1 death per 100,000 persons-years).

**Fig. 2:**
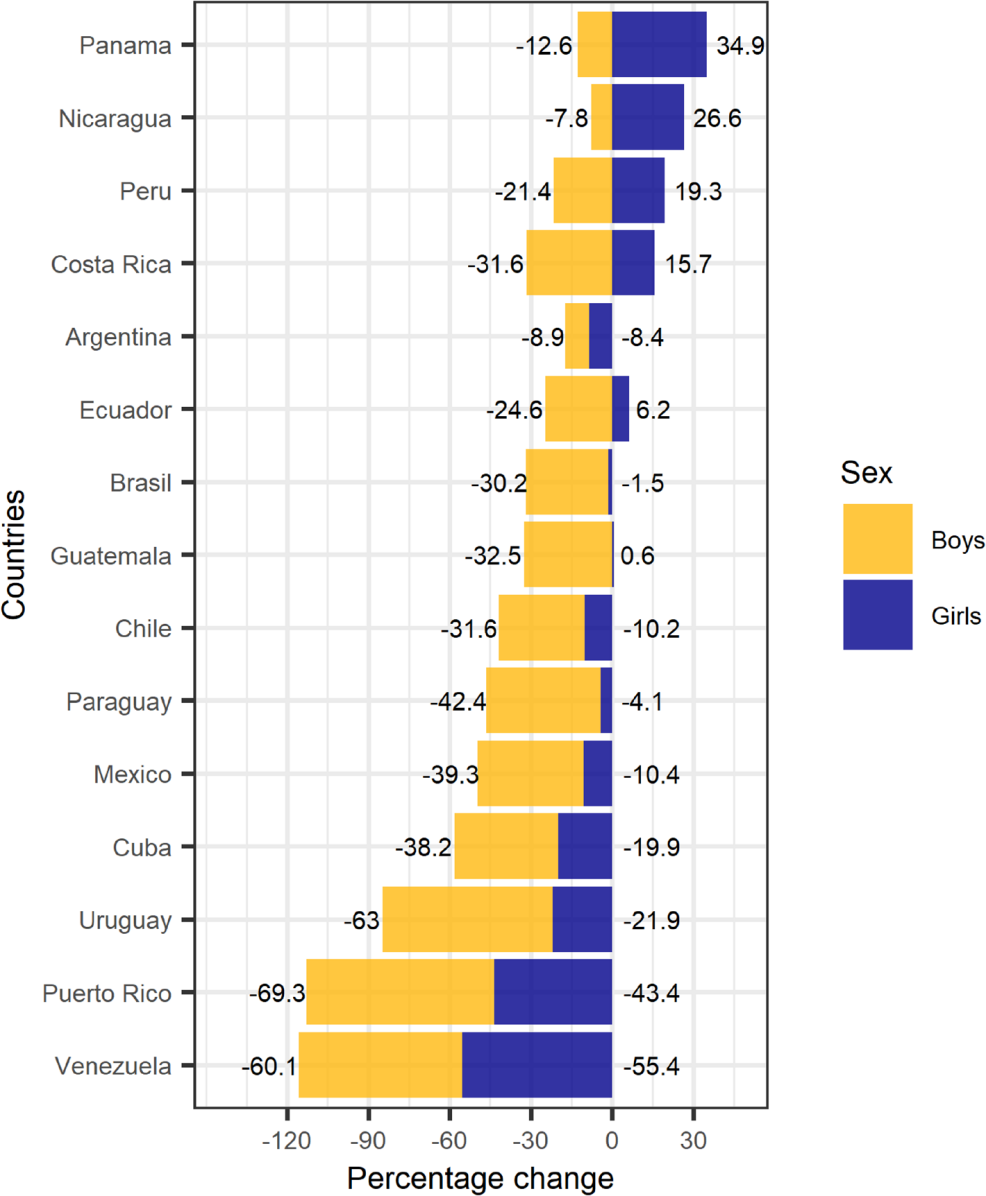
Percent changes between the periods 2000–2005 and 2012–2017 in Latin American countries

Figure 2 illustrates the percent changes between the periods 2000–2005 and 2012–2017 in LAC countries. Most countries reported decreases, mainly in boys. The greatest decreases were reported in Venezuela (− 60% in boys and − 55% girls), Puerto Rico (− 69% in boys and − 43% in girls), and Uruguay (− 63% in boys and − 22% in girls). Some differences by sex were reported in Panama, Nicaragua, Peru, Costa Rica, Ecuador, and Guatemala. For instance, Panama declined by 12.6% among boys, but increased by 34.9% among girls, Nicaragua declined by 7.8% among boys, but increased by 26.6% among girls, and Peru declined by 21.4% among boys, but increased 19.3% among girls (See Supplementary 1).

**Fig. 3:**
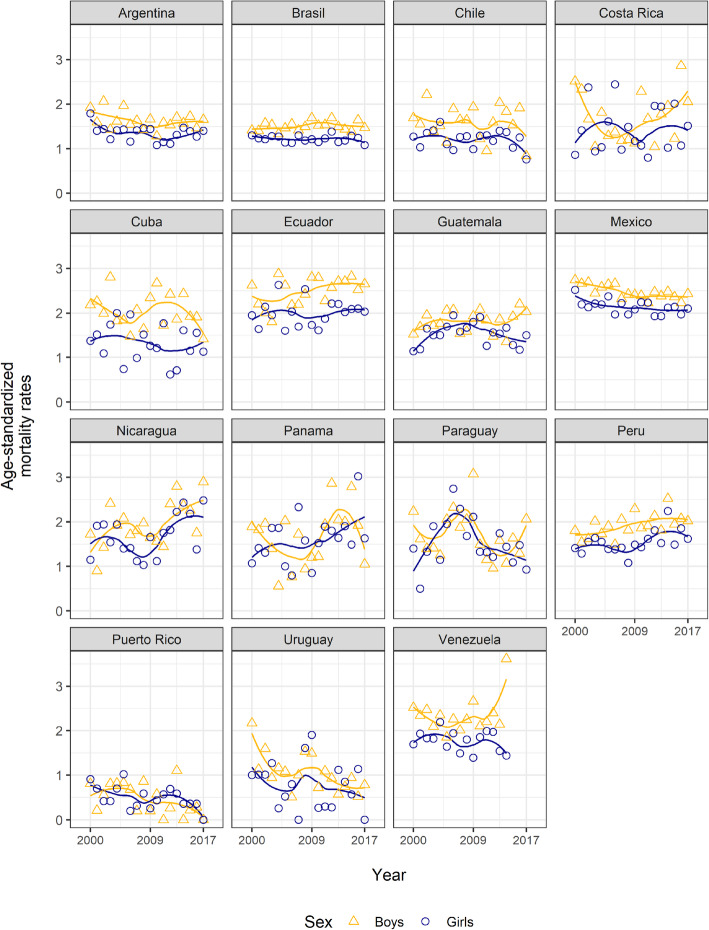
Leukemia mortality trends in children from 15 Latin American countries 2000–2017

**Fig. 4:**
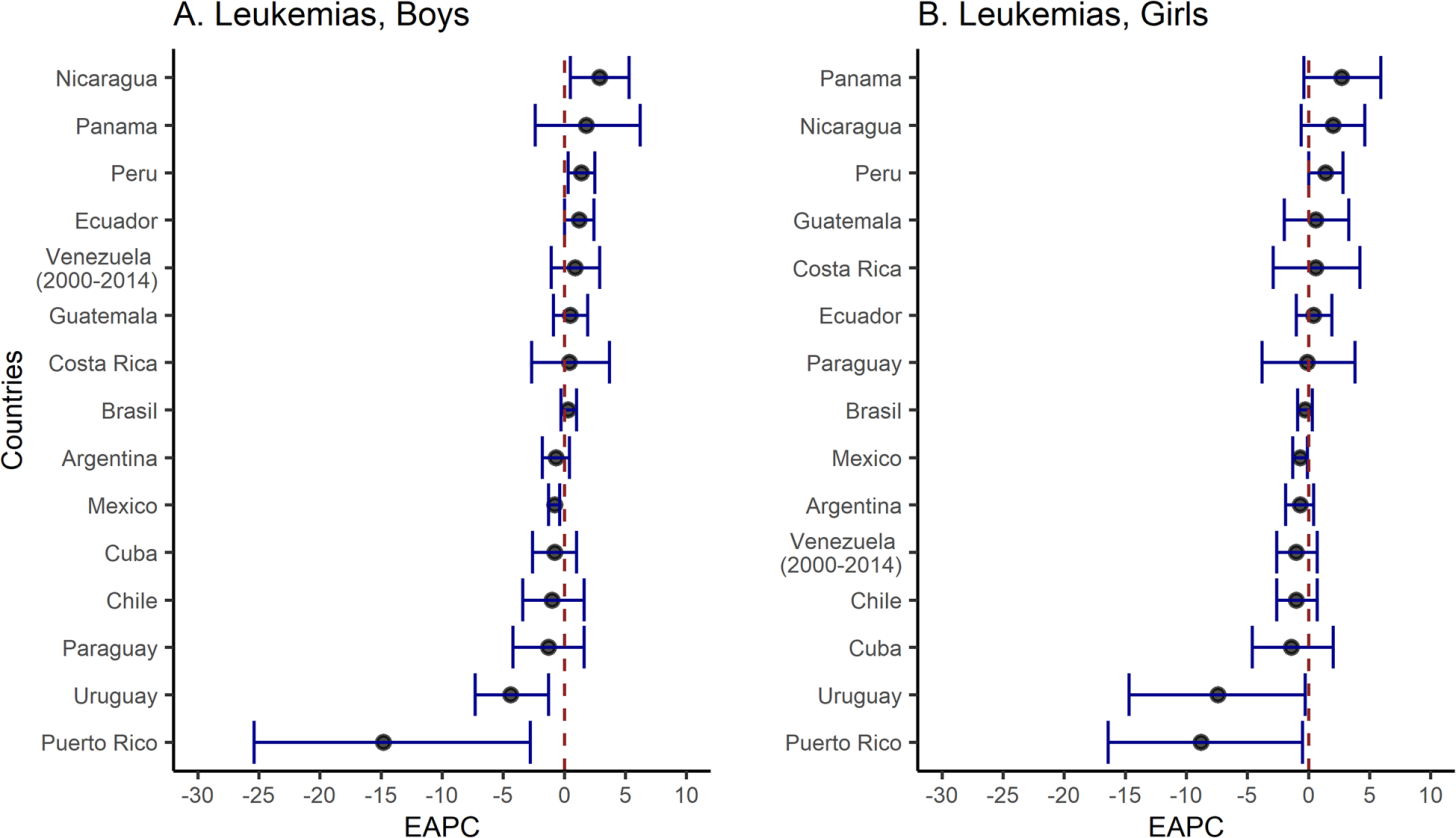
Estimated annual percent change (EAPC, %) and 95% confidence interval (CI) for leukemia mortality rates among children in Latin America and the Caribbean

In boys, Nicaragua (EAPC = 2.9, 95% CI: 0.5, 5.3, *p* < 0.05) and Peru (EAPC = 1.4, 95% CI: 0.3, 2.5, *p* < 0.05) had significant upward trends in whole period, whereas Puerto Rico (EAPC = − 14.8, 95% CI: − 25.4, − 2.8, *p* < 0.05) and Uruguay (EAPC = − 4.4, 95% CI: − 7.3, − 1.3, *p* < 0.05) experienced downward trends. Whereas in girls, only Peru had an upward trend (EAPC = 1.4, 95% CI: 0.1, 2.8, p < 0.05), while, three countries showed downward trends, Puerto Rico (EAPC = − 8.8, 95% CI: − 16.4, − 0.5, p < 0.05) experienced the greatest reduction, followed by Uruguay (EAPC = − 7.4, 95% CI: − 14.7, − 0.3, p < 0.05) and Mexico (EAPC = − 0.7, 95% CI: − 1.3, − 0.1, p < 0.05) (Fig. 3, Fig. 4, and Supplementary 2).

Table 1 shows the number of projected leukemia deaths in boys, age-standardized mortality rates and percentage change in deaths due to population and risk between 2015 and 2030. The forecast indicates that mortality rates will increase in Argentina, Brazil, Chile, Ecuador, Guatemala, Mexico, Peru, Puerto Rico, and Uruguay; and decline in Costa Rica, Cuba, Nicaragua, Panama, and Paraguay. The evaluation of change due to risk between 2015 and 2030, among boys, found a pronounced increase in Argentina (+ 21.8%), Uruguay (+ 29.8%), and Ecuador (+ 31.8%). The predicted total reduction in rates in Puerto Rico (− 56.7%) would be predominantly due to population change (− 58.7%), despite an increase in the risk of leukemia mortality (+ 2.1%). Ecuador is forecast to have an increase in population (+ 5.8%) and risk of death (+ 31.8%), resulting in an overall increase for leukemia mortality (+ 37.6%).

**Table 1:** Number of leukemia death in boys, age-standardized mortality rates and percentage change in cases due to population and risk, 2015 and 2030

Countries	Population (per million)		Number of deaths		Age-standardized mortality rates		Total change (%)	Change due to population (%)	Change due to risk (%)
	2015	2030	2015	2030	2015	2030			
Argentina	5.5	5.6	450	554	1.61	1.91	23.0	1.2	21.8
Brazil	24.4	20.9	1813	1645	1.48	1.57	−9.3	−14.3	5.0
Chile	1.8	1.6	153	146	1.64	1.69	−4.6	−9.0	4.4
Costa Rica	0.5	0.5	55	43	1.97	1.70	−21.1	−8.0	−13.1
Cuba	1.0	0.8	87	65	1.91	1.70	−25.9	−15.3	−10.6
Ecuador	2.3	2.5	326	449	2.68	3.44	37.6	5.8	31.8
Guatemala	3.0	3.1	288	313	1.87	1.93	8.8	5.2	3.5
Mexico	17.8	16.2	2164	2031	2.38	2.45	−6.2	−9.0	2.8
Nicaragua	0.8	0.9	114	115	2.42	2.32	0.5	2.3	−1.8
Panama	0.5	0.6	53	56	1.96	1.80	5.1	13.4	−8.3
Paraguay	1.0	1.1	82	74	1.52	1.40	−9.4	0.9	−10.3
Peru	4.4	4.2	465	490	2.07	2.27	5.4	−4.9	10.3
Puerto Rico	0.4	0.2	6	3	0.32	0.34	−56.7	−58.7	2.1
Uruguay	0.4	0.4	13	16	0.71	0.93	23.1	−6.7	29.8

Among girls, mortality rates will potentially increase in most LAC countries. In addition, there will be an increase in the risk of leukemia mortality in Argentina, Chile, Cuba, Ecuador, Nicaragua, Peru, Puerto Rico, and Uruguay which was the basis for the predicted increase among all the countries (except for a decline in Chile of 4.9%). For Cuba, and Puerto Rico, there will a reduction in mortality resulting from changes in population structure and size, whereas in Brazil, Costa Rica, and Mexico there will be a reduction in mortality due to both population changes and a decrease in the risk of leukemia mortality (Table 2).

**Table 2:** Number of leukemia deaths in girls, age-standardised mortality rates and percentage change in cases due to population and risk, 2015 and 2030

Countries	Population (per million)		Number of new deaths		Age-standardised mortality rates		Total change (%)	Change due to population (%)	Change due to risk (%)
	2015	2030	2015	2030	2015	2030			
Argentina	5.3	5.4	369	419	1.37	1.51	13.5	1.4	12.1
Brazil	23.4	19.9	1370	1109	1.18	1.14	−19.0	−15.0	−4.0
Chile	1.8	1.6	103	98	1.16	1.22	−4.9	−9.4	4.6
Costa Rica	0.5	0.4	41	32	1.51	1.29	−22.2	−7.6	−14.5
Cuba	0.9	0.8	55	49	1.23	1.28	−11.3	−14.5	3.3
Ecuador	2.3	2.4	241	295	2.09	2.40	22.3	5.2	17.1
Guatemala	2.9	3.1	211	206	1.43	1.35	−2.6	4.3	−6.9
Mexico	17.2	15.5	1784	1545	2.05	1.99	−13.4	−9.5	−3.8
Nicaragua	0.8	0.9	96	141	2.14	2.91	47.1	3.1	43.9
Panama	0.5	0.6	52	51	1.94	1.71	−2.5	10.7	−13.2
Paraguay	0.9	1.0	62	51	1.29	1.05	−17.3	6.6	−23.8
Peru	4.3	4.1	378	507	1.74	2.40	34.1	−4.8	38.9
Puerto Rico	0.3	0.1	5	3	0.34	0.42	−40.0	−53.1	13.1
Uruguay	0.4	0.3	13	14	0.73	0.83	6.2	−7.0	13.2

## Discussion

This study provides a comprehensive population-based analysis of mortality trends for leukemia among children in 15 LAC countries. We found that Peru and Nicaragua had significant upward trends from 2000 to 2017, while most of the remaining countries showed little variation in mortality trends. Moreover, the analysis identified a wide range of mortality rates among LAC countries in the last 5 years of the study period (from 0.32 to 2.88 in boys and from 0.33 to 2.14 in girls).

Puerto Rico and Uruguay had significant downward trends in both sexes, along with the lowest mortality rates in the region. These outcomes are in line with the mortality trends worldwide [5, 9]. A study of southern and eastern European countries found a significant decrease in mortality rates of childhood leukemia [7]. Similarly, Bertuccio et al. [5] found downward leukemia mortality trends in the United States, Japan, and Western and Central European countries [5], which could be explained by the improvement of healthcare delivery and the development and implementation of novel treatment regimens [5, 27, 28].

Mortality rates for childhood leukemia in many LAC countries remain higher than those in high-income countries [11, 29]. Our study shows mortality rates up to 2.88 in boys and up to 2.14 in girls, whereas European countries report mortality rates up to 1.63 among boys and up to 1.35 among girls [5]. Another study in the United States estimated a mortality rate of 0.71 for both sexes from 2007 to 2010 [6]. Few countries in our analysis have mortality rates as low as those of the United States and European nations for both sexes. In the last 5 years of our study (2013–2017), Venezuela, Ecuador, and Nicaragua reported the highest mortality rates among boys and Nicaragua, Ecuador, and Mexico among girls. Previous studies also reported higher mortality rates for Mexico, Ecuador, and Venezuela than other LAC countries [9, 10]. For this reason, it is necessary to identify risk factors for leukemia mortality in further research in these countries. Another relevant finding is the high mortality rates among children from Venezuela, which could be related to the country’s socioeconomic and political instability in recent years [30], limiting the healthcare delivery to the pediatric population. Additionally, we only estimated mortality rates until 2014, due to a lack of national information on cancer deaths reports for the 2015–2017 period, restraining a proper comparison with the remaining LAC countries.

Important gaps remain in LAC countries to reduce the mortality of childhood leukemia, such as socioeconomic inequalities, low access to high-quality health care, delayed diagnosis, and limited access to novel treatment [29, 31–33]. Studies have shown a relationship between childhood leukemia and factors related to economic status, mainly in low-income African countries [29, 34], and that these economic disparities affect access to health services in certain communities within countries [32, 35]. However, childhood leukemia and economic status do not appear to be significantly related in high-income countries such as Switzerland or the US [31, 36], suggesting that others factors have contribute to leukemia mortality. For example, some reports found that abandonment of treatment is a major barrier to successful treatment and remission of the disease in low- and middle-income countries [14, 37, 38], which could increase the mortality rates in this population. Although these inequities are plausible explanations for the unremarkable trends seen in our analysis, there is still the need to explore further factors such as environmental hazard or components of the healthcare system, to generate tailored and evidence-based health policies.

The comparison between changes resulting from the increase in leukemia mortality and changes resulting from demographic population structure are notable. Ecuador and Argentina showed a higher risk of death for boys, and Nicaragua and Peru for girls. The predicted rates in Peru may not be attained however as, the World Health Organization chose Peru in 2018 as the Index Country to accomplish a 60% increase in survival of Pediatric Cancer in 2030 [39], where the Ministry of Health and stakeholders are committed to working toward that goal.

Possible problems related to the increased risk of leukemia mortality may be related to social inequalities and access to health systems as mentioned above [29, 31–33]. In addition, countries such as Puerto Rico had a decline in the mortality due to changes in population size and structure, even with an increased risk of leukemia mortality. The changes associated with the population structure may be related to factors that differ by country, such as a reduction in the birthrate. Studies that assess trends over time allow the creation and improvement of public policies aimed at a better structuring of the health system, based on predictions for the future. Moreover, they allow health planning, especially for the most vulnerable groups [40, 41].

A limitation of this study is the variability of the data records of each country and the lack of availability of data for some countries such as Honduras, Belize, and Bolivia. Secondly, we could not analyze incidence data given the lack of population-based cancer registries in most countries. Nevertheless, our study gives the most recent comprehensive epidemiological analysis of mortality patterns for childhood leukemia from LAC countries and provides a forecast to 2030, the target year by which WHO aims to improve childhood cancer survival to least 60%. The results of this study should prompt further research in areas such as the relationship between socioeconomic status, healthcare delivery, or environmental hazard and mortality or incidence of childhood leukemia.

## Conclusions

Overall, we found a wide variation in mortality trends among children under 15 years of age in most LAC countries. Furthermore, LAC nations should perform interventions to reduce socioeconomic inequalities and ensure universal healthcare coverage to prevent the increasing mortality rates projected by 2030.

## Supplementary Information

**Additional file 1: Supplementary 1**. Age-standardized mortality rates from leukemia in children from Latin America and the Caribbean in the periods 2000–2005, and 2012–2017, and corresponding percent changes.

**Additional file 2: Supplementary 2**. Estimated annual percent change (EAPC, %) and 95% confidence interval (CI) for leukemia mortality rates in 0–14 years.

## Data Availability

The datasets generated and/or analysed during the current study are available in the following link: https://www.who.int/healthinfo/statistics/mortality_rawdata/en/

## Abbreviations

ASMRs: Age-standardized mortality rates
LAC: Latin American and the Caribbean
