# Supplementary material for: Leukemia mortality in children from Latin America: trends and predictions to 2030
Source: BMC Pediatr. 2020 Nov 7;20:511. doi: 10.1186/s12887-020-02408-y (PMC7648388; doi:10.1186/s12887-020-02408-y)
Supplement: Supplementary file 1 — Additional file 1: Supplementary 1. Age-standardized mortality rates from leukemia in children from Latin America and the Caribbean in the periods 2000–2005, and 2012–2017, and corresponding percent changes. [file 12887_2020_2408_MOESM1_ESM.doc]

**Supplementary 1. Age-standardized mortality rates from leukemia in children from Latin America and the Caribbean in the periods 2000–2005, and 2012-2017, and corresponding percent changes.**

| **Countries** | **Boys** | | | **Girls** | | |
| --- | --- | --- | --- | --- | --- | --- |
|  | 2000-05 | 2012-17 | %change 2012-17/2000-05 | 2000-05 | 2012-17 | %change 2012-17/2000-05 |
| **Argentina** | 1.76 | 1.61 | −8.9 | 1.45 | 1.33 | −8.4 |
| **Brazil** | 2.18 | 1.52 | −30.2 | 1.24 | 1.22 | −1.5 |
| **Chile** | 2.37 | 1.62 | −31.6 | 1.29 | 1.16 | −10.2 |
| **Costa Rica** | 2.66 | 1.82 | −31.6 | 1.37 | 1.59 | 15.7 |
| **Cuba** | 3.22 | 1.99 | −38.2 | 1.41 | 1.13 | −19.9 |
| **Ecuador** | 3.53 | 2.66 | −24.6 | 1.99 | 2.11 | 6.2 |
| **Guatemala** | 2.68 | 1.81 | −32.5 | 1.44 | 1.45 | 0.6 |
| **Mexico** | 3.94 | 2.39 | −39.3 | 2.27 | 2.03 | −10.4 |
| **Nicaragua** | 2.61 | 2.41 | −7.8 | 1.65 | 2.09 | 26.6 |
| **Panama** | 2.41 | 2.11 | −12.6 | 1.42 | 1.91 | 34.9 |
| **Paraguay** | 2.49 | 1.43 | −42.4 | 1.37 | 1.32 | −4.1 |
| **Peru** | 2.65 | 2.08 | −21.4 | 1.47 | 1.76 | 19.3 |
| **Puerto Rico** | 1.01 | 0.31 | −69.3 | 0.7 | 0.39 | −43.4 |
| **Uruguay** | 2.02 | 0.75 | −63.0 | 0.85 | 0.66 | −21.9 |
| **Venezuela** | 3.41 | 1.36 | −60.1 | 1.85 | 0.83 | −55.4 |
