# Supplementary material for: Leukemia mortality in children from Latin America: trends and predictions to 2030
Source: BMC Pediatr. 2020 Nov 7;20:511. doi: 10.1186/s12887-020-02408-y (PMC7648388; doi:10.1186/s12887-020-02408-y)
Supplement: Supplementary file 2 — Additional file 2: Supplementary 2. Estimated annual percent change (EAPC, %) and 95% confidence interval (CI) for leukemia mortality rates in 0–14 years. [file 12887_2020_2408_MOESM2_ESM.doc]

**Supplementary 2. Estimated annual percent change (EAPC, %) and 95% confidence interval (CI) for leukemia mortality rates in 0-14 years.**

| Country | Boys | Girls |
| --- | --- | --- |
|  | EAPC (95% CI) | EAPC (95% CI) |
| Argentina | −0.7(−1.8, 0.4) | −0.7(−1.9, 0.4) |
| Brazil | 0.3(−0.3, 1.0) | −0.3(−0.9, 0.3) |
| Chile | −1.0(−3.4, 1.6) | −1.0(−2.6, 0.7) |
| Costa Rica | 0.4(−2.7, 3.7) | 0.6(−2.9, 4.2) |
| Cuba | −0.8(−2.6, 1.0) | −1.4(−4.6, 2.0) |
| Ecuador | 1.2(0.0, 2.4) | 0.4(−1.0, 1.9) |
| Guatemala | 0.5(−0.9, 1.9) | 0.6 (−2.0, 3.3) |
| Mexico | −0.8(−1.3, −0.4) | −0.7(−1.3, −0.1) |
| Nicaragua | 2.9(0.5, 5.3) | 2.0(−0.6, 4.6) |
| Panama | 1.8(−2.4, 6.2) | 2.7(−0.4, 5.9) |
| Paraguay | −1.3(−4.2, 1.6) | −0.1(−3.8, 3.8) |
| Peru | 1.4(0.3, 2.5) | 1.4(0.1, 2.8) |
| Puerto Rico | −14.8(−25.4, −2.8) | −8.8(−16.4, −0.5) |
| Uruguay | −4.4*(−7.3, −1.3) | −7.4(−14.7, −0.3) |
| Venezuela | 0.9(−1.1, 2.9) | −1.0(−2.6, 0.7) |
